# Incidence of first ever stroke during Hajj ceremony

**DOI:** 10.1186/1471-2377-13-193

**Published:** 2013-12-05

**Authors:** Mahmoud Reza Azarpazhooh, Reza Bavarsad Shahripour, Moira K Kapral, Naghmeh Mokhber, Ali Shoeibi, Mohammad Taghi Farzadfard, Mohammad Reza Rafati, Amanda G Thrift, Negar Morovatdar, Seyed Aidin Sajedi, Amir Azarpazhooh

**Affiliations:** 1 Department of Neurology, Ghaem Hospital, Mashhad University of Medical Sciences, Mashhad, Iran; 2 Comprehensive Stroke Center, University of Alabama Hospital, Birmingham, AL, USA; 3 Department of Neurology, Golestan Hospital, Ahvaz University of Medical Sciences, Ahwaz, Iran; 4 Institute of Health Policy, Management and Evaluation, Faculty of Medicine, University of Toronto, Ontario, Canada; 5 Department of Neuropsychology, Ibn-e-Sina Hospital, Mashhad University of Medical Science, Mashhad, Iran; 6 Department of Internal Medicine, Red Crescent Society, Tehran, Iran; 7 Department of Medicine, Southern Clinical School, Monash University, Melbourne, and National Stroke Research Institute, Florey Neuroscience Institutes, Heidelberg Heights, Victoria, Australia; 8 Health System Research Committee, Treatment Affairs of vice chancellery, Mashhad University of Medical Sciences, Mashhad, Iran; 9 Discipline of Dental Public Health, Faculty of Dentistry, University of Toronto, Room 515-C, 124 Edward St, Toronto, ON M5G 1G6, Canada; 10 Toronto Health Economics and Technology Assessment Collaborative, University of Toronto, Toronto, Canada

**Keywords:** Acute stroke, Hajj, Incidence

## Abstract

**Background:**

The Hajj Ceremony, the largest annual gathering in the world, is the most important life event for any Muslim. This study was designed to evaluate the incidence of stroke among Iranian pilgrims during the Hajj ceremony.

**Methods:**

We ascertained all cases of stroke occurring in a population of 92,974 Iranian pilgrims between November 27, 2007 and January 12, 2008. Incidence and risk factors of the first ever stroke in Hajj pilgrims were compared, within the same time frame, to those of the Mashhad residents, the second largest city in Iran. Data for the latter group were extracted from the Mashhad Stroke Incidence Study (MSIS) database.

**Results:**

During the study period, 17 first-ever strokes occurred in the Hajj pilgrims and 40 first-ever stroke strokes occurred in the MSIS group. Overall, the adjusted incidence rate of first ever stroke in the Hajj cohort was lower than that of the MSIS population (9 vs. 16 per 100,000). For age- and gender-specific subgroups, the Hajj stroke crude rates were in general similar to or lower than the general population of Mashhad, Iran, with the exception of women aged 35 to 44 years and aged >75 years who were at greater risk of having first-ever stroke than the non-pilgrims of the same age.

**Conclusion:**

The first ever stroke rate among Iranian Hajj pilgrims was lower than that of the general population in Mashhad, Iran, except for females 35–44 or more than 75 years old. The number of events occurring during the Hajj suggests that Islamic countries should consider designing preventive and screening programs for pilgrims.

## Background

The Hajj Ceremony, the largest annual gathering in the world, is the most important life event for any Muslim. For the majority of the pilgrims, this is a long-desired journey that may only happen once in their lifetime. Hence, it can be mentally stressful. Annually, millions of Muslims from various countries gather in the holy cities of Mecca and Medina in the Kingdom of Saudi Arabia to perform their religious rituals. Pilgrims are often in close contact, and are at increased risk of viral infections, particularly upper respiratory tract infections [1-5].

Despite the importance of Hajj, there are scant data on the incidence of important diseases such as vascular conditions that occur during the pilgrimage. Quantifying rates of cardiovascular disease during Hajj is important for Islamic countries [6-9]. If the stroke rate is high during Hajj, the Kingdom of Saudi Arabia may need to re-evaluate its health system infrastructure and hospitals for stroke treatment. Moreover, Islamic countries may need to develop vascular preventive and screening programs for pilgrims. In addition, because of high reported rates of infection and psychological stress, the Hajj pilgrimage brings a unique opportunity to evaluate such risk factors on the occurrence of stroke [6].

### Objective

The objectives of this study were: 1) to determine the incidence of first-ever stroke among Iranian pilgrims during the Hajj ceremony, and 2) to compare the stroke incidence rate to a proxy estimate of Iran’s national stroke rate.

## Methods

### Study design and source of data

The study was approved by the Ethics Committee of the Hajj and Pilgrimage Organization of Iran and Mashhad University of Medical Sciences (protocol No. 2195390). The study participants were 92,974 Iranian pilgrims who attended the Hajj ceremony between November 27, 2007 and January 12, 2008. These pilgrims were from different socioeconomic backgrounds and different rural and urban areas. According to the guidelines of the Hajj and Pilgrimage Organization of Iran, candidates with severe dementia, uncontrollable cancer, severe disabling stroke and recent (less than 3 months) myocardial infarction are not eligible to participate in the pilgrimage. After the initial eligibility screening, pilgrims were gathered as 605 groups (caravans, each including 100–200 pilgrims) and were evaluated before, during, and after the Hajj ceremony. Before and during the trip, a General Practitioner (GP) was assigned to each caravan.

Before the trip, the GPs received extensive training in the medical protocol of the Hajj and Pilgrimage Organization of Iran. They were responsible for registering past medical conditions, including the history of previous stroke, and evaluating the health status eligibility of the pilgrims in their assigned caravans. This information was recorded in the patient health chart kept by the GP of each caravan. During the Hajj pilgrimage, the GPs were the first point of contact for the pilgrims in case of medical concerns. The GPs then referred cases with neurological findings to the two permanent hospitals of the Iran Red Crescent Society in the Holy cities of Mecca and Medina, under the care of the study neurologist (MRA). When imaging was required, the cases were referred to the local Saudi's Hospitals. All patients were provided with free access to hospital care and laboratory tests. After the Hajj ceremony, all Iranian pilgrims were fully covered by a private healthcare company for any follow up visits, admission and treatment of diseases related to the Hajj period. The health data of the pilgrims, including their demographic characteristics, past medical history, medical events during and after the Hajj ceremony, decisions taken by GPs and subsequent referrals, cause of admission and outcome were registered in the central data bank of the Hajj and Pilgrimage Organization of Iran. We received the de-identified dataset and ascertained all cases of first ever-stroke in Hajj pilgrims through the retrospective evaluation of pilgrims’ medical records. We also ascertained stroke events in non-pilgrims from the Mashhad Stroke Incidence Study (MSIS), our recently published population-based prospective cohort study [10]. The MSIS registered all strokes occurring in a population of 450,229 residents of the City of Mashhad (the second largest city in Iran) were registered during a 12-month period (2006–2007) [10].

### Variables and outcome measures

The primary outcome in this study was the incidence of stroke, defined according to the World Health Organization as rapidly developing signs of focal or global disturbance of cerebral function, lasting for more than 24 hours (unless interrupted by surgery or death) with no apparent cause other than a vascular origin [11,12]. The First-Ever Stroke was defined as a stroke occurring for the first time during a patient’s lifetime [12]. Previous stroke was determined using all available information including hospital records, neuroimaging results and/or self- or family-reported data. Neuroimaging was used to classify cases with definite strokes into ischemic stroke, intra-cerebral haemorrhage, or subarachnoid haemorrhage. As per the most recent consensus definition the American Heart Association/American Stroke Association (AHA/ASA), we included cerebral venous thrombosis (CVT) as stroke only if patients had infarction or hemorrhage in localized area of brain because of thrombosis of a cerebral venous structure [12].

An “undetermined stroke” was defined as a stroke in which a patient had not undergone CT scanning within 28 days of the onset of symptoms and an autopsy had not been performed [12].

A “possible stroke” was considered as any acute episode of neurological disturbance that was suggestive of stroke but there was insufficient information to establish whether or not the symptoms and duration (<24 hours or > 24 hours) fully met the World Health Organization definition of the definite stroke [13]. A “CT only” stroke was defined when the patient had no clinical signs and symptoms of stroke, but neuroimaging identified changes compatible with stroke [13]. “Possible” and “CT only” strokes were excluded from the study. We also excluded CVT cases for whom the symptoms or signs were caused by reversible edema without infarction or hemorrhage [12].

The following risk factors were assessed for those patients diagnosed with first-ever stroke: gender, age, stressful periods (i.e., mentally or emotionally disruptive or upsetting situations such as getting lost, losing money, missing ritual rules, etc.), current smoking status, and past history of hypertension, diabetes, hyperlipidaemia, transient ischemic attack, ischemic heart disease, and atrial fibrillation. For our second objective, we regarded the Hajj pilgrims as the exposed group and compared them to the non-exposed population, derived from the data source of the MSIS stroke incidence study, in the manner of a historical cohort. From the MSIS database, the first-ever stroke cases during the exact period of the Hajj ceremony were extracted.

### Statistical analysis

The first-ever stroke crude incidence rates were calculated using the total of Iranian Hajj pilgrims and the MSIS subjects (above age of 15, in the same time frame), respectively as the denominator for the Hajj group and the MSIS group. To calculate standardized incidence rates, we used direct method of adjustment with SEGI world population and IRAN 2006 standard population. We calculated the adjusted incidence rates for each population and by gender. Due to the different population pyramids of Hajj pilgrims and Mashhad residents, the crude incidence rates were compared only for stratified age and gender groups between two cohorts. The 95% confidence intervals (CI) were calculated for adjusted rates on the assumption of a normal distribution for counts greater than 100 and a Poisson distribution for counts less than 100. The incidence rates were reported as number of cases per 100,000 population per month. Proportions were compared by the Chi-square test or Fisher's exact test when appropriate. Students T-test was used to compare continuous variables. All statistical tests were undertaken using SPSS v13.0 software. The significance was set at P < 0.05 (two-sided).

## Results

### Descriptive results

**Figure 1:**
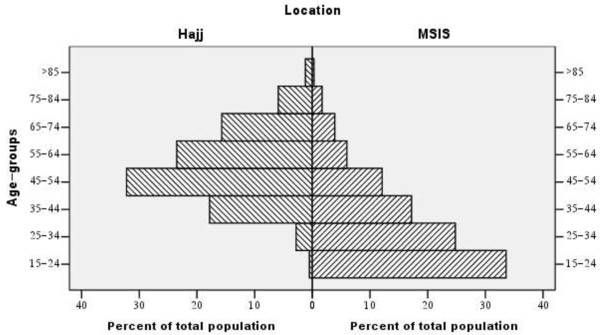
Distribution of Hajj and MSIS cohorts by age groups.

The age distribution of the Hajj pilgrims and the MSIS population is presented in Figure 1. While 55% of the MSIS population was less than 35 years of age, the majority of the pilgrims (75%) were between the ages of 35 and 64 years. During the study period, GPs referred 19 cases suspected of having stroke. A final diagnosis of first-ever stroke by the study neurologist was made for 17 of these cases (5 men and 12 women). No case was suspected of “possible” or “CT only” strokes. Thirteen patients had ischemic stroke (including two cases with cerebral vein thrombosis); two had intra-cerebral haemorrhage; and the other two had undetermined stroke. CT or MRI of the brain was performed in 15 cases. Within the same time period, 40 cases (23 men and 17 women) with a final diagnosis of first-ever stroke were registered in the MSIS database. These included 29 cases of ischemic stroke, 10 cases of haemorrhagic stroke and one case of undetermined stroke. The baseline characteristics and risk factors of first-ever stroke patients were similar between the two groups (Table 1).

**Table 1:** Age- and gender -specific Incidence Rates (per 100,000 Population per month) and adjusted incidence rates for First-Ever Stroke in Hajj (2007) compared with the data from MSIS within the same period

**Age (years)**	**Hajj subjects**			**MSIS subjects**			**P value**
	**No.**	**Population at risk**	**Incidence rate (95% CI)**	**No.**	**Population at risk**	**Incidence rate (95% CI)**	
**Both genders**							
15–24	0	514	–	0	112588	–	–
25–34	0	4110	–	2	83131	2 (0–5)	–
35–44	2	19397	10 (4–16)	1	57743	2 (0–5)	0.021^‡^
45–54	3	29973	10 (4–16)	5	40596	12 (5–20)	0.67^†^
55–64	3	21584	14 (7–21)	5	20096	25 (15–36)	0.07^†^
65–74	5	12568	40 (28–52)	11	13103	84 (66–102)	0.001^†^
75–84	3	4033	74 (57–91)	13	5944	219 (190–248)	0.001^†^
>85	1	739	135 (112–158)	3	1147	262 (230–294)	0.001^†^
**Total**	17	92974	18 (10–26)	40	334348	12 (5–19)	
Standardized to SEGI*			7 (4–11)			13 (9–17)	0.03
**Standardized to IRAN ^**			7 (5–9)			12 (8–15)	0.04
**Males**							
15–24	0	234	–	0	55458	–	–
25–34	0	1303	–	1	42266	2 (0–5)	–
35–44	0	8102	–	0	29202	–	–
45–54	2	14646	14 (7–21)	3	20681	15 (7–23)	0.85^†^
55–64	2	10704	19 (10–28)	2	10146	20 (11–29)	0.87^†^
65–74	0	7140	–	5	6820	73 (56–90)	–
75–84	1	2710	37 (25–49)	10	3109	322 (287–357)	0.001^†^
>85	0	547	–	2	556	360 (323–397)	–
**Totals**	5	45419	11 (4–18)	23	168238	14 (7–21)	
Standardized to SEGI*			4 (0.5–7)			14 (8–20)	0.02
**Standardized to IRAN ^**			4 (0.5–8)			17 (10–24)	0.01
**Females**							
15–24	0	280	–	0	57130	–	–
25–34	0	2807	–	1	40865	2 (0–5)	–
35–44	2	11295	18 (10–26)	1	28541	4 (0–8)	0.003^†^
45–54	1	15327	7 (2–12)	2	19915	10 (4–16)	0.46^†^
55–64	1	10880	9 (3–15)	3	9950	30 (19–41)	0.001^†^
65–74	5	5428	92 (73–111)	6	6283	95 (76–114)	0.82^†^
75–84	2	1323	151 (127–175)	3	2835	106 (86–126)	0.005^†^
>85	1	192	521 (476–566)	1	591	169 (144–194)	0.001^†^
**Total females**	12	47555	25 (15–35)	17	166110	10 (4–16)	
Standardized to SEGI*			13 (6–21)			11 (6–17)	0.07
**Standardized to IRAN^**			15 (6–23)			12 (7–18)	0.08

MSIS: Mashhad Stroke Incidence Study [11].

^†^Chi-square test; ‡Fisher exact test; N/A: Not applicable.

*adjusted by age and sex to the world (SEGI) population.

^adjusted by age and sex to the Iran population.

### First-ever stroke incidence

Table 1 summarizes the age- and gender- specific crude incidence rates as well as the SEGI- and IRAN 2006- adjusted rates of first-ever stroke in the two cohorts. For the age- and gender- specific crude incidence rates, we noted a lower first-ever stroke rates in the Hajj group for all strata, except for women aged 35 to 44, 75 to 84 and greater than 85 years. In these subgroups, stroke rates were significantly higher in the Hajj than in the MSIS cohort. Overall, the SEGI-adjusted incidence rate in the Hajj cohort was lower than that of the MSIS population (7; 95% CI: 4–11 vs. 13; 95% CI: 9–17 per 100,000 per month; P = 0.03). This pattern was similar for males (4; 95% CI:0.5-7 vs.14; 95% CI: 8–20, per 100,000 per month ; P = 0.02). However, the adjusted incidence rate of first-ever stroke in the Hajj female population was not statistically different from the MSIS population (13; 95% CI: 6–21 vs. 11; 95% CI:6–17 per 100,000 per month; P = 0.07). The IRAN 2006-adjusted rates followed a pattern similar to those of the SEGI-adjusted rates.

## Discussion

We investigated the incidence of stroke among Iranian pilgrims during the 2007 Hajj ceremony, and found that the overall incidence was lower as compared to what was seen in the general population of Mashhad, Iran. This pattern is likely attributable to the pre-pilgrimage medical screening performed by the Hajj and Pilgrimage Organization of Iran. The screening program for the Hajj is simple, comprising evaluation of blood pressure, diabetes and other vascular risk factors. In suspected cases of vascular disorders, electrocardiography is performed with referral for cardiology or neurology consultation. Candidates with severe dementia, uncontrollable cancer, severe disabling stroke and recent (less than 3 months) myocardial infarction are excluded from participation in the Hajj ceremony. Therefore, it can be argued that such enhanced screening system may have reduced to some degree the stroke risk factors by selecting individuals devoid of severe and life-threatening disease. The finding implies a research hypothesis whether population-based screening programs would reduce stroke in communities. This simple screening could be tested in the general population with the aim of treating these risk factors to prevent vascular events. It is also possible that the pilgrims with the knowledge of having the upcoming trip would be more conscious about their health status. In other words, people participating in the Hajj are healthier than the general population, similar to what is observed in the healthy migrant effect [14].

Previous small-scale studies reported that 20-35% of hospital admissions during Hajj were related to old age and occurred in patients with associated cardiovascular conditions [15-18]. To the best of our knowledge no study has thus far assessed the incidence of stroke among the Hajj participants. In this present study, we found that stroke risk in Hajj pilgrims was increased relative to the general population of Mashhad, Iran in some subgroups of women, including those aged 33 to 44 years and those aged over 75 years. We have previously reported a greater risk of CVT in Hajj pilgrim females [18-38]. Young Muslim women often take oral contraceptives in the short term during religious ceremonies to avoid menstruation. This is because menstruation would cancel the sacred state of Ihram, into which a Muslim must enter in order to perform the religious rituals. In this present study, we had two cases with CVT who fell in the stroke criteria of the AHA/ASA [12]. It is possible that the use of oral contraceptives during the Hajj contributed to the increased risk of stroke in women aged 35 to 44 years compared to the general population of Mashhad, Iran. It needs to be noted that some epidemiological studies of stroke did not include CVT. Ischemic stroke and CVT are important types of stroke and comply with the clinical definition of stroke. In 2013 Updated Definition of Stroke for the 21st Century, the American Heart Association/American Stroke Association included CVT with specific criteria (infarction or hemorrhage in brain because of thrombosis of a cerebral venous structure) as stroke [9]. Therefore, we included these two cases that fell under these specific criteria.

In addition, pilgrims are subjected to high rates of physical and mental stress as well as infection, in particular upper respiratory tract infections) [19]. Previous studies have suggested that recent bacterial or viral infections, particularly in the preceding week, are a risk factor for stroke [19-21]. Some studies have also shown an association between respiratory tract infection epidemics and death due to cardiovascular disorders [19], and reductions in cardiovascular mortality and stroke after influenza vaccination [22-24] and control of epidemic respiratory diseases [25-27]. While such infections, inflammatory processes and psychological stress may be predisposing risk factors for stroke and cardiovascular disease, their exact role remains unclear [28-33].

Within the Hajj pilgrims, an increased risk of first-ever stroke was noted in females aged over 65 years compared to their male counterparts. The reasons for this are uncertain, and may include baseline differences in stroke risk factors or comorbid conditions. Other potential explanations include gender differences in the neurobiological responses to psychological stress. It has been shown that brain locations activated in response to psychological stress, the subsequent systemic responses and the duration of the effect of the stress are different in men and women [22,34,35]. That said, the existing literature regarding psychological distress and risk of stroke is scant, and presents varied findings [22-36].

This study has some limitations. Any occurrence of stroke would have health planning implications; however, in order to have a comparable group with the MSIS subjects, we evaluated only first ever strokes rather than all strokes. Although the pre-pilgrimage medical screening may have resulted in overall lower first-ever stroke rates in Iranian pilgrims, it may have imposed an unavoidable selection bias, once comparing the Hajj pilgrims with the general population of Mashhad, Iran. The latter group comprises those with diverse socio-economical status and underlying factors that may affect their health. Mashhad is the second populous metropolitan of Iran and a religious centre that attracts many migrants from all parts of the country. The MSIS has been so far the only comprehensive population-based study of stroke in Iran. Although the result of this study cannot readily be generalized to the whole country, other unpublished local data suggest comparable cerebrovascular risk profile in other cities, similar to what was found in the MSIS. This gives us some confidence that Mashhad can be regarded as representative of the urban population of Iran and the MSIS results can provide a good proxy of the national incidence of urban population, but not the rural population. It should also be noted that the baseline information and risk profile were only available from the Hajj pilgrims with first-ever stroke, not all the pilgrims. Therefore, the comparison between demographic and pre-morbid risk factors of all Hajj pilgrims and the general population of Mashhad, Iran was not possible. In addition, we were unable to evaluate the processes and outcomes of care for Hajj pilgrims with stroke.

Despite these limitations, this study was strengthened by the fact that we accessed the medical information of more than 90,000 Iranian pilgrims who were closely monitored during the Hajj ceremony. To the best of our knowledge, this is the first stroke epidemiological study among Hajj pilgrims, gathering at the largest annual event in the world. Islam has 1.6 billion followers in the world, comprising over 23% of the world population [39]. Due to the religious rules, attending in Hajj ceremony, at least for once, is an aim for Muslims. Iran is one of the 22 Member States of the WHO Eastern Mediterranean Region (EMRO). These Member States, with a population of nearly 583 million people, are predominantly Muslims [40]. Therefore, the findings of this study may be considered of value for the EMRO countries in preparation for the Hajj ceremony, a very large population.

## Conclusions

In conclusion we found that the first ever stroke rate among Iranian Hajj pilgrims was lower than that of the general population in Mashhad, Iran, except for females 35–44 or more than 75 years old. Although the results cannot be generalized to pilgrims from other countries, they highlight the importance of preventive and screening programs aimed to reduce vascular risk factors, stress, and respiratory infections, and the need for the Kingdom of Saudi Arabia to evaluate the infrastructure of its healthcare system and hospitals to ensure that appropriate medical care including acute stroke treatment is available during the Hajj.

## Competing interests

The authors declare that they have no competing interests.

## Authors’ contributions

MRA has made substantial contributions to the study conception, design, and implementation. He revised the manuscript critically for important intellectual content. RBS participated in the study design and has been involved in drafting and revising the manuscript critically for important intellectual content. MKK and AGT helped in revising the manuscript critically for important intellectual content according to its result. NM and MRR helped to design and coordinate the study. AS and MTF participated in the study design and data collection. SAS and NMD performed the statistical analysis and has been involved in drafting the manuscript. AA has made substantial contributions to conception and design, helped to plan for statistical analysis, and has been involved in drafting the manuscript and revising it critically for important intellectual content. All authors read and approved the final manuscript.

## Pre-publication history

The pre-publication history for this paper can be accessed here:

http://www.biomedcentral.com/1471-2377/13/193/prepub

## References

[B1] Greenwood DC Muir KR Packham CJ Madeley RJ Coronary heart disease: a review of the role of psychosocial stress and social support J Public Health Med 1996 18 2 221 231 Epub 1996/06/01 10.1093/oxfordjournals.pubmed.a024483 8816321

[B2] Nieto FJ Infections and atherosclerosis: new clues from an old hypothesis? Am J Epidemiol 1998 148 10 937 948 Epub 1998/11/26 10.1093/oxfordjournals.aje.a009570 9829865

[B3] Grau AJ Buggle F Heindl S Steichenwiehn C Banerjee T Maiwald M Recent infection as a risk factor for cerebrovascular ischemia Stroke 1995 26 3 373 379 10.1161/01.STR.26.3.373 7886709

[B4] Grau AJ Buggle F Steichenwiehn C Heindl S Banerjee T Seitz R Clinical and biochemical-analysis in infection-associated stroke Stroke 1995 26 9 1520 1526 10.1161/01.STR.26.9.1520 7660391

[B5] Mattila KJ Valtonen VV Nieminen MS Asikainen S Role of infection as a risk factor for atherosclerosis, myocardial infarction, and stroke Clin Infect Dis 1998 26 3 719 734 Epub 1998/04/03 10.1086/514570 9524851

[B6] Meysamie A Ardakani HZ Razavi SM Doroodi T Comparison of mortality and morbidity rates among Iranian pilgrims in Hajj 2004 and 2005 Saudi Med J 2006 27 7 1049 1053 16830029

[B7] Jakovljevic D Salomaa V Sivenius J Tamminen M Sarti C Salmi K Seasonal variation in the occurrence of stroke in a finnish adult population. The FINMONICA stroke register. Finnish monitoring trends and determinants in cardiovascular disease Stroke 1996 27 10 1774 1779 Epub 1996/10/01 10.1161/01.STR.27.10.1774 8841328

[B8] Everson SA Roberts RE Goldberg DE Kaplan GA Depressive symptoms and increased risk of stroke mortality over a 29-year period Arch Intern Med 1998 158 10 1133 1138 Epub 1998/05/30 10.1001/archinte.158.10.1133 9605786

[B9] Simonsick EM Wallace RB Blazer DG Berkman LF Depressive symptomatology and hypertension-associated morbidity and mortality in older adults Psychosom Med 1995 57 5 427 435 Epub 1995/09/01 8552732 10.1097/00006842-199509000-00003

[B10] Azarpazhooh MR Etemadi MM Donnan GA Mokhber N Majdi MR Ghayour-Mobarhan M Excessive incidence of stroke in iran: evidence from the Mashhad stroke incidence study (MSIS), a population-based study of stroke in the Middle East Stroke 2010 41 1 e3 e10 Epub 2009/11/21 10.1161/STROKEAHA.109.559708 19926844

[B11] Colantonio A Kasl SV Ostfeld AM Depressive symptoms and other psychosocial factors as predictors of stroke in the elderly Am J Epidemiol 1992 136 7 884 894 10.1093/aje/136.7.884 1442754

[B12] Sacco RL Kasner SE Broderick JP Caplan LR Connors JJ Culebras A An updated definition of stroke for the 21st century: a statement for healthcare professionals from the American heart association/American stroke association Stroke 2013 44 7 2064 2089 Epub 2013/05/09 10.1161/STR.0b013e318296aeca 23652265 PMC11078537

[B13] Marnane M Duggan CA Sheehan OC Merwick A Hannon N Curtin D Stroke subtype classification to mechanism-specific and undetermined categories by TOAST, A-S-C-O, and causative classification system: direct comparison in the North Dublin population stroke study Stroke 2010 41 8 1579 1586 Epub 2010/07/03 10.1161/STROKEAHA.109.575373 20595675

[B14] Fennelly K The "healthy migrant" effect Minn Med 2007 90 3 51 53 Epub 2007/04/17 17432759

[B15] Al-Ghamdi SM Akbar HO Qari YA Fathaldin OA Al-Rashed RS Pattern of admission to hospitals during muslim pilgrimage (Hajj) Saudi Med J 2003 24 10 1073 1076 Epub 2003/10/28 14578971

[B16] W.H A The prevalence of cardiovascular disease and role of protective measures among Hajj pilgrims 1432 Pak J Pharmacol 2012 29 29 34

[B17] Khan NAIA Ahmad MS El-Sayed FM Bachal ZA Abbas TG Pattern of medical diseases and determinants of prognosis of hospitalization during 2005 Muslim pilgrimage Hajj in a tertiary care hospital. A prospective cohort study Saudi Med J 2006 27 1373 1380 16951776

[B18] Azarpazhooh MR Rafi S Etemadi MM Khadem N Fazlinejad A The relation between short-term oral contraceptive consumption and cerebrovascular, cardiovascular disorders in Iranian women attending Hajj Saudi Med J 2008 29 7 1024 1027 18626534

[B19] Alzeer AH Respiratory infection in haj ceremony Ann Thorac Med 2009 4 50 53 10.4103/1817-1737.49412 19561924 PMC2700482

[B20] Yamamoto Perceived mental stress and mortality from cardiovascular disease among japanese men and women: the Japan collaborative cohort study for evaluation of cancer risk sponsored by monbusho Circulation 2002 106 1229 1236 10.1161/01.CIR.0000028145.58654.41 12208798

[B21] Andre-Petersson L Engstrom G Hagberg B Janzon L Steen G Adaptive behavior in stressful situations and stroke incidence in hypertensive men - Results from prospective cohort study "men born in 1914" in Malmo, Sweden Stroke 2001 32 8 1712 1717 10.1161/01.STR.32.8.1712 11486095

[B22] Harmsen P Rosengren A Tsipogianni A Wilhelmsen L Risk factors for stroke in middle-aged men in Goteborg, Sweden Stroke 1990 21 2 223 229 Epub 1990/02/01 10.1161/01.STR.21.2.223 2305396

[B23] Macko RF Ameriso SF Barndt R Clough W Weiner JM Fisher M Precipitants of brain infarction. Roles of preceding infection/inflammation and recent psychological stress Stroke 1996 27 11 1999 2004 Epub 1996/11/01 10.1161/01.STR.27.11.1999 8898805

[B24] WassertheilSmoller S Applegate WB Berge K Chang CJ Davis BR Grimm R Change in depression as a precursor of cardiovascular events Arch Intern Med 1996 156 5 553 561 10.1001/archinte.1996.00440050111012 8604962

[B25] Zeller JA LA Eschenfelder CC Zunker P Deuschl G Platelet–leukocyte interaction and platelet activation in acute stroke with and without preceding infection Arterioscler Thromb Vasc Biol 2005 25 1519 1523 10.1161/01.ATV.0000167524.69092.16 15845906

[B26] Laurila A Bloigu A Nayha S Hassi J Leinonen M Saikku P Chronic Chlamydia pneumoniae infection is associated with a serum lipid profile known to be a risk factor for atherosclerosis Arterioscl Throm Vas 1997 17 11 2910 2913 10.1161/01.ATV.17.11.2910 9409275

[B27] Mayr M Kiechl S Willeit J Wick G Xu Q Infections, immunity, and atherosclerosis: associations of antibodies to Chlamydia pneumoniae, Helicobacter pylori, and cytomegalovirus with immune reactions to heat-shock protein 60 and carotid or femoral atherosclerosis Circulation 2000 102 8 833 839 Epub 2000/08/23 10.1161/01.CIR.102.8.833 10952949

[B28] Housworth J Langmuir AD Excess mortality from epidemic influenza, 1957–1966 Am J Epidemiol 1974 100 1 40 48 Epub 1974/07/01 4858301 10.1093/oxfordjournals.aje.a112007

[B29] Alling DW Blackwelder WC Stuart-Harris CH A study of excess mortality during influenza epidemics in the United States, 1968–1976 Am J Epidemiol 1981 113 1 30 43 Epub 1981/01/01 7457477 10.1093/oxfordjournals.aje.a113063

[B30] May MMP Stansfeld S Ben-Shlomo Y Gallacher J Yarnell J Does psychological distress predict the risk of ischemic stroke and transient ischemic attack? The Caerphilly study Stroke 2002 33 7 12 10.1161/hs0102.100529 11779881

[B31] Bova IY Bornstein NM Korczyn AD Acute infection as a risk factor for ischemic stroke Stroke 1996 27 12 2204 2206 10.1161/01.STR.27.12.2204 8969781

[B32] Grau AJ Buggle F Becher H Zimmermann E Spiel M Fent T Recent bacterial and viral infection is a risk factor for cerebrovascular ischemia - Clinical and biochemical studies Neurology 1998 50 1 196 203 10.1212/WNL.50.1.196 9443480

[B33] House A Dennis M Mogridge L Hawton K Warlow C Life events and difficulties preceding stroke J Neurol Neurosurg Psychiatr Suppl 1990 53 12 1024 1028 Epub 1990/12/01 10.1136/jnnp.53.12.1024 PMC488307 2292691

[B34] Wang J Korczykowski M Rao H Fan Y Pluta J Gur RC Gender difference in neural response to psychological stress Soc Cogn Affect Neurosci 2007 2 3 227 239 Epub 2007/09/18 10.1093/scan/nsm018 17873968 PMC1974871

[B35] Andre-Petersson L Engstrom G Hagberg B Janzon L Rosvall M Social support at work and the risk of myocardial infarction and stroke in women and men Soc Sci Med 2007 64 4 830 41 Epub 2006 Nov 22 10.1016/j.socscimed.2006.10.020 17123677

[B36] Bainton D Jones GR Hole D Influenza and ischaemic heart disease–a possible trigger for acute myocardial infarction? Int J Epidemiol 1978 7 3 231 239 Epub 1978/09/01 10.1093/ije/7.3.231 721358

[B37] Saidee MFM Sasannejad P Mellat Ardakani A Azarpazhooh M The relation between short course oral contraceptive consumption and cerebral vein thrombosis in Ramadan Iran J Neurol 2008 7 23 260 523

[B38] Sasannejad PAA Velayati A Shoeibi A Saeidi M Foroughipour M Cerebral vein thrombosis in women using short course oral contraceptive consumption Iran J Reprod Med 2012 10 6 537 542 25246923 PMC4169846

[B39] The Global Religious Landscape A Report on the Size and Distribution of the World’s Major Religious Groups as of 2010 2012 Washington, D.C: Pew Research Center’s Forum on Religion & Public Life Available from: http://www.pewforum.org/files/2012/12/globalReligion-full.pdf

[B40] World Health Organization Regional Office for the Eastern Mediterranean (EMRO) http://www.emro.who.int/entity/about-us/index.html 10.26719/2012.18.4.325 22768693

